# Supplementary material for: Patterns of Intron Gain and Loss in Fungi
Source: PLoS Biol. 2004 Nov 30;2(12):e422. doi: 10.1371/journal.pbio.0020422 (PMC532390; doi:10.1371/journal.pbio.0020422)
Supplement: Table S1 — Also available at http://genes.mit.edu/NielsenEtAl/. (4.3 MB ZIP). [file pbio.0020422.st001.zip › NielsenEtAl/html/827.html]

AN8838.1.NCU00095.1.MG06462.1.FG06948.1


```
 CLUSTAL W (1.82) Multiple Sequence Alignments - Introns Inserted


Sequence 1: NCU00095.1	1279 aa
Sequence 2: MG06462.1	1221 aa
Sequence 3: FG06948.1	1219 aa
Sequence 4: AN8838.1	1467 aa
Alignment Length: 1719 aa
Number Identitical Residues: 465 aa
Alignment Score (without introns) 26145


MG06462.1 	------------------------------------------------------------
NCU00095.1	------------------------------------------------------------
FG06948.1 	------------------------------------------------------------
AN8838.1  	MSESELRQRKPAARLRDDPQPSQDAQPRLKHGIPMQVLRSLLLATWFNCCCVVILMTQLI
          	 :.:.  . ..::   .....:..:..  . . . .   :   ::  ....     :.  

MG06462.1 	------------------------------------------------------------
NCU00095.1	------------------------------------------------------------
FG06948.1 	------------------------------------------------------------
AN8838.1  	GCPLYIINKHYYYAWMASTKRSFGLVITSLTEWGCPTYVRVSGDESIRGQVRIADDGRLK
          	...    ..    :  :::. : .   :: :. ...:    :...:  ..   :...  .

MG06462.1 	------------------------------------------------------------
NCU00095.1	------------------------------------------------------------
FG06948.1 	------------------------------------------------------------
AN8838.1  	TTFPERLVMISNHQVYTDWIYLWWIAYSNMMHGHIFIILKESLKYIPIIGQGMTFYGFIF
          	:: ..     :. .  :.       : :.   .      ..: .  .  ... :  .   

MG06462.1 	------------------------------------------------------------
NCU00095.1	------------------------------------------------------------
FG06948.1 	------------------------------------------------------------
AN8838.1  	MARKWLSDKPRLQHRLEKLKTQHIGSDSGAPKYDPMWLLIFPEGTNLSINTKRRSDAYGA
          	 : .  :...  .   .. .:.  .:.:.:.. ..      ...:. : .:.  :.: .:

MG06462.1 	-------------------------------------------~----------------
NCU00095.1	-------------------------------------------~----------------
FG06948.1 	-------------------------------------------~----------------
AN8838.1  	KNGFPPLKHQVLPRSTGLFFCLQQLRGTVEWVYDCTVGYEGPP2KGSYADKYFTLRSTYV
          	... .. . .  . ::.   . ..  .: .   ..: . .... ..: :..  :  ::  

MG06462.1 	------------------------------------------------------------
NCU00095.1	------------------------------------------------------------
FG06948.1 	------------------------------------------------------------
AN8838.1  	QGRPPTSVNMYWRRFAVSDIPLDDQQEFDAWLRARWTEKDELLDEYFETGRFPSALAGSI
          	.. ..:: .      : :. . ..... .:   :  :....  ..  .:.  .:: :.: 

MG06462.1 	-----------------------------~-----------------------~------
NCU00095.1	-----------------------------~-----------------------~------
FG06948.1 	-----------------------------~-----------------------~------
AN8838.1  	EVGHGDAQQLNAAEDGYAEAHVRLGHWTE0VYDGMLLYLNNSPCLLSDRGTWK0DLLLLS
          	. . ..:.. .::... :.:    .  :.   ..     ..:..  :. .: . .    :

MG06462.1 	-------------MASSNRNGNVN-------GSAAAG---HPIS-----KRFSDVPPSID
NCU00095.1	-------------MASAALNGAAANGRGVSPASSVGGSNSFPKS-----KRFTDIPSTID
FG06948.1 	--------------MSSSQNSTTN--------GVNGDAQSAPVS-----TRFSDIPSAID
AN8838.1  	STELLSPRLEGGVAGSRSLRTPAMTSLRNKYSNGIVNGDSLPVTGVLANSRFSDIPSAID
          	::.  :.  ...   *   .  . ..   . ..   ...: * :.  :..**:*:*.:**

MG06462.1 	VPVQD--EDEAVEINLEDLVDDPTDLCDLFENENAARTYWMAVALAYAKLHKIDCAIEVL
NCU00095.1	IPVQDQ-DDEAVEIDLEVLADDPTELCTLFEMEGAARTYWMTVSLAYAKQKKIDFAIEML
FG06948.1 	IPIPSGEADEAVEINLEDLVDEPADLCTLLENENAAKTYWMTVALAYAKQHKVDHAIEML
AN8838.1  	IPAST--LDSEVEVSLEGLPDDPTELCTLLENERAAKNFWVIIALAYAKQKQIDHAIDIL
          	:*      *. **:.** * *:*::** *:* * **:.:*: ::***** :::* **::*

MG06462.1 	TRGASAMQNH--NPREKLGIVSCLCWMYLWKSREAPRVAPEGALASEVKTKEHYLQLANT
NCU00095.1	IKGANAMQAN--NPREKLSIVSALCWMYLWKSREAPRVAPEDALVSEAKTKEYYLQLATQ
FG06948.1 	QRGGNEIQKNSANPRDRVSMVCCLCWMYLWKSREAPRVAPEGTPVSEAKTKEYYLQLATS
AN8838.1  	NKGLASVAQG--ATKEKLGLLGWVCWLYLLKSREAPRVVSDVGLGTEARTKDYYIQQATG
          	 :*   :      .::::.::  :**:** ********..:    :*.:**::*:* *. 

MG06462.1 	TFNEASRINPAFPPLFLAKGVLYLLRASLQTSVKSGQVGSVDAAKAGLLGSALKSFEDAL
NCU00095.1	SLNEASRINPAFPPLFLARGVLQLLRASLQPPSKAP--GAVDPEKTETLRAALKSFDDAL
FG06948.1 	SLNDAARLNPSFPPIFLARGVLLLLRASLQAPSKTA--GGIGSEKHELLKTAVKSFDDAL
AN8838.1  	ILNEASRLNPSFPPLFLARGVLSLLRASLHPPRPVRPGAVDNSERVESLRQALKQFDESS
          	 :*:*:*:**:***:***:*** ******:..      .  .. :   *  *:*.*::: 

MG06462.1 	RVSQGKNMLALMGKSRAQFSLGKYAEALAGYQDVLHRMPEMVDPDPRLGIGCCFWQLGHK
NCU00095.1	RVSSGKNMLAVIGKARALFSLGRYADALVAYQDALARAPDLVDPDPRIGIGCCFWQLGYK
FG06948.1 	RVSQGKNMLALMGKARALFSMHKYPESLTIYQDVLQKMPDLVDPDPRIGIGCCFWQLGFK
AN8838.1  	KAFGGRNAMAILGRARAHYLLGRYAEALEGYQKVLMRMPGLTDPDPRIGIGCCLWQLGFK
          	:.  *:* :*::*::** : : :*.::*  **..* : * :.*****:*****:****.*

MG06462.1 	DDAKAAWERCLEI~NPESKIANILVGLYYLDQSGQVPTNSPEFIRLYKKAMTEYTQKSFK
NCU00095.1	DDARIAWERALEI~NAESKVGNILLGLYYLDASGHVPINSPEFIKLYKKAMTEYTQKSYK
FG06948.1 	DDAKMAWERCLEI~NPNSKVANILLGLYYLDASGHVPVNSDNFLKLYKKAMTEYAQKSFK
AN8838.1  	EQAKAAWERALAL0NPDSKVANILLAVYYLYDSSRHATTDPAFGSLYKVAMTQYTQKAFK
          	::*: ****.* : *.:**:.***:.:***  *.: . ..  *  *** ***:*:**::*

MG06462.1 	LSKDLPLTCATFAGYFLSRKSFANVDSLAHKAIQYTDVNAIASDGWYLLARKEHYQGDTE
NCU00095.1	LDKDLPLTCATFASYFLSRKQFDHVEALAHKAIQFTDVNAIASDGWYLLARKEHYANNLE
FG06948.1 	LDKDVPLTCSTFSGYFLSRKAWDNVDKLAHKAIQYTDVNAIASDGWYLLARKEHYTGDIE
AN8838.1  	LDKEYPMTCALFGSYFLLRKAYSTVDTLARKSIENTDVMQIASDGWYLLGRKCHYEGDLA
          	*.*: *:**: *..*** ** :  *: **:*:*: ***  *********.** ** .:  

MG06462.1 	KASDYYRRSDDARGGTDRGYLPAKFGVAQLSVVKEDYGEAKLRLEKMIQQSRNYEAMILL
NCU00095.1	RAADYYRRADDARGGTERGYLPAKFGAAQLSVIKNDLGEAKLRLEKMIQQSKNYEAMILL
FG06948.1 	RAADYYRRADEARGGTETGYLPAKFGVAQLSVMKNDLGEAKLRLEKMIQQSKNHEAMILL
AN8838.1  	KAAEFYHRSDQARGGGDKGYLPAKFGSVQMQVSNKDYDGAKFQLEKIIQQTKNPECMTLL
          	:*:::*:*:*:**** : ******** .*:.* ::* . **::***:***::* *.* **

MG06462.1 	GTLYAEEVFATQYANVKEDKSAEIKKAISLLEGVRTAWKDTKKNLSPDAAVLLNLARLYE
NCU00095.1	GNLYAEEVFANQFAPVKEDKSAEAKKAISLLESVRTAWKDPKKSLAPDAAVLLNLARLYE
FG06948.1 	GTLYAEEVFANQVSDSKEDKSAELKKAITLLESVRNAWKDPKRALSPDASVLLNLARLYE
AN8838.1  	AALYAEEVFAAQKSGMKEDKSAEAKKAITLFEAVRALWKDESKKITPDESVLVYLSRLYE
          	. ******** * :  ******* ****:*:*.**  *** .: ::** :**: *:****

MG06462.1 	TEHPDKALQCLQQVEQLEIELISDSDRPQGIEDEAQLKAAVRKLLPPQLLNNIGCFHAQH
NCU00095.1	TENPDKALQCLQQVEQLELDQVPQSERPDEVEGEAAIKAALRRFLPPQLLNNIGCFYYQE
FG06948.1 	TDQPDKALQCLQQVEQLELDQVPDSEQPEDVTDAAEIKAALRKSLPPQLLNNIGCFHSQA
AN8838.1  	QNAPDKSMQCLTQLEELQLAEISDEERPEGLEDE-EMKAALRVNLPPQLLNNMGCFLYQS
          	 : ***::*** *:*:*::  :.:.::*: : .   :***:*  ********:***  * 

MG06462.1 	ERHEAASDMFEAALGACMRIQESDDQMDTDALVTTISFNLGRSYESRGLWDQAIEVYEGL
NCU00095.1	EKHELASELFEAALSSCMRIGEKDDDTDTDALVTTISFNLGRSYEARGMSEKAVEVYEGL
FG06948.1 	EKHELASDMFEAALGACIKIGEKDPDMDTDALVSTISFNLARSYESRGLTDKATEVYEGL
AN8838.1  	SQLNMARSMFQAALDSCARSQEREGELDTDALVTTISYNLGRAFEASDMPDEAKKVYEAL
          	.: : * .:*:***.:* :  * : : ******:***:**.*::*: .: ::* :***.*

MG06462.1 	LKRHDDYTDAKIRLAYIKLRRNP-QKEGPDAVAKLYQGNPADLEVRALYGWYLGKVHSRK
NCU00095.1	LKRHDDYTDARTRLAYIKLRNNPGTKEGPDAVAKLYQENSSDLEVRALYGWFLGKLSSRK
FG06948.1 	LARHDDYTDARARLAYIKLRKNP-NKEGPDAVAKLYQENNTDLEVRALYGWYLGKVHSRK
AN8838.1  	LERHSDYTEASARLTYLALRRSP-TDEGPKKMAKLYETDSTNLEVRALFGWYLSK--SKK
          	* **.***:*  **:*: **..*  .***. :****: : ::******:**:*.*  *:*

MG06462.1 	RPANIAEDPELRHYKHTLQHYDKHDCYALVGMGNLYLMSAREMRRETDQDKSRRSATYSR
NCU00095.1	RPNNIAEDPEQRHYKHTLQNYDKHDRYALVGMGNLHLISAREMRRESEADRQKRSAAYSR
FG06948.1 	RPANLNEDAEFRHYKHTLQNYDKHDRHALVGMGNLYLMQAREMRRESDSDKQKRSAIYAK
AN8838.1  	RAANIAEDHEQRHHKHTLQYFDKHDRYALTGMGNVHLLFARDMRRDTDQEKEKRRKMYER
          	*. *: ** * **:***** :**** :**.****::*: **:***::: ::.:*   * :

MG06462.1 	AVEFFEKALQLDPRNAYAAQGIAIALVEDRKDHKSALQVFLSVRDTIKDAQVLVNLGHIF
NCU00095.1	AVEFFDKALQLDPKNAYAAQGIAIALVEDKKDYKGALQIFIKVRETIKDVHVFVNLGHIY
FG06948.1 	AVEFFEKALSLDPKNAYAAQGIAIALVEDKKDHKSALGIFNKVRETLRDSHLYVNLGHIF
AN8838.1  	AVEFFDKALQLDPRNAYAAQGIAIALVDDRKDLSTAVQIFSKIRDSLRDASVYLNLGHVY
          	*****:***.***:*************:*:** . *: :* .:*::::*  : :****::

MG06462.1 	AELKQFTKAIENYEAALAKEGKANDPSILACLGRTWLNKGRAERDLEAYMKALDCAKK0A
NCU00095.1	AELKQFTKAIESYEIALGKEGKAKDANILSCLGRTWLNKGRAERNLDAYKTALEYAQK0T
FG06948.1 	AELRQYSKAIEHYEIALSKDGKKDDPTILACLGRTWLNRGRAERDVDSYNKALECAQK0A
AN8838.1  	AELRQYTRSIEHYEAALSKD-RARDAQILACLGRVWLLKGKQEMSLAAMKTALDYAQR~A
          	***:*::::** ** **.*: :  *. **:****.** :*: * .: :  .**: *:: :

MG06462.1 	LNVAPEQVHFKFNVAFVQIQLATTIYGLSETQRSLEQLQDAATGLESAIAALDELAAHPQ
NCU00095.1	LEVAPEQVHFKFNVAFVQIQLASFINGLPEHQRTSTQLEEAASGLESAITALDEIAASDH
FG06948.1 	LGVAPEQVHYKFNVAFVQIQLVTLVQGLPENKRSTEQLEKAAEGLESAITSLDEIAAHPQ
AN8838.1  	HSVAPTQAHLEFNVAFVQNQIASLTYSLPETQRTAQDVQDAADGLRTAVETFGRIAQAKN
          	  *** *.* :******* *:.:   .*.* :*:  :::.** **.:*: ::..:*   :

MG06462.1 	TPYPKQDVEQRANMARNTQRKQLERAIASQKEYEEKNKEKLAAALELRQAALKKREEEKQ
NCU00095.1	PPYPKHDIEQRANMARNTQRKQLERALASQRDYESKNKEKLQLALEQRQVALKKKEEEIR
FG06948.1 	TPYPKNDVEQRANMARNTLRNQLQRAIGKQKEWEEKNKEKIQAAKELREAEVRRREEERQ
AN8838.1  	PPYPAESLEQRANMSK-TIIKQLERALQSQKEYEEKNAAKLQQAREAREAEIRRREEEVR
          	.*** ..:******:: *  :**:**: .*:::*.**  *:  * * *:. ::::*** :

MG06462.1 	KAAEAERERQEKIRKEREAIAARDRELAEARHREQQAKEEAEMTTDGETGEKIKR-KRKA
NCU00095.1	RKEEEERERQEKIKREREEIAARDRKLAEQRAEEDRQRREAEMTTDSETGEKIKRQKIKK
FG06948.1 	KIVELERERQEKIKKERDEIAKKDRALAAQRAEEEQARMEAEMTTDSETGDKVKR-KRKT
AN8838.1  	KAEEAERERKQRIAEERQRLVEEAQRLALS-------------------GQKRNG-----
          	:  * ****:::* .**: :. . : **                     *:* :      

MG06462.1 	AGGGGGK---SGESRGAKGARSSRKKKP--TTDDDQSDVEDSEAEQ-----RQPKKKRRL
NCU00095.1	ASSTPAKRERGGEERERKGRAQRKKKTSRRDRDNDDSDGSGSDAEEGGRKSRPPKKKQRL
FG06948.1 	APR------ASGEPKPKRSGGGKKKKKS--EHQDDESE----EEEE------RTKKRRRL
AN8838.1  	-----------------------------------------------------PAKKRR-
          	                                                     . *::* 

MG06462.1 	TQNKKVDNTKYKSAEIVVDSDEDEE---EYDALDRAERKLD--DSRLSTP------DSDL
NCU00095.1	TSRKTEPTGKYKSAEIVVDSDESDEPGMEMDALERAERALEKKQKRSHSPRSGGEYDDDE
FG06948.1 	TKKE---SAKFKSAEIVVDSDEENNN--DDDELERAERNID----REETP----------
AN8838.1  	------------------------------------------------------------
          	                                                            

MG06462.1 	GEPAG-GRQSDEE--DRMDVDKS----GGGDGDDDDDATNSRPKRSRRGGRVLDESDDED
NCU00095.1	DEQSRRGRSRSEESVDRMEIDDSKADAGGDDEEEEATVSTRRQNKRSRRGRILDDDDDED
FG06948.1 	--LSEIDEKVVED--DKMDVDGG--AEQGGDDDDEEEIAPRQQNKRARRGRVVSDEEDND
AN8838.1  	------------------------------------------------------------
          	                                                            

MG06462.1 	ED----GTPAEK---------------GTIAADTKMGDAEEDEE
NCU00095.1	EDEDESGAAAEADAEEGGDKDEVKEAAGSPAASPAKAAANDDEE
FG06948.1 	DEEAPTQAKTASGDEDD---------EPRPAADTSMADAADDEE
AN8838.1  	--------------------------------------------
          	
```
